# Rhythmic sampling and competition of target and distractor representations in visual sensory memory

**DOI:** 10.1093/cercor/bhag018

**Published:** 2026-02-24

**Authors:** Sean Noah, Sreenivasan Meyyappan, Mingzhou Ding, George R Mangun

**Affiliations:** Department of Neuroscience, University of California, Berkeley, 1 Barker Hall, Berkeley, CA 94720, United States; Center for Mind and Brain, University of California, Davis, 267 Cousteau Place, Davis, CA 95618, United States; J. Crayton Pruitt Family Department of Biomedical Engineering, University of Florida, 1275 Center Drive, Biomedical Science Bldg. J285, Gainesville, FL 32611, United States; Center for Mind and Brain, University of California, Davis, 267 Cousteau Place, Davis, CA 95618, United States; Department of Psychology, University of California, Davis, 1 Shields Ave, Davis, CA 95616, United States; Department of Neurology, University of California, Davis, 4860 Y Street, Sacramento, CA 95817, United States

**Keywords:** attention, behavior, EEG, decoding, rhythmic sampling

## Abstract

Recent studies suggest that sustained visual attention operates rhythmically, as if the visual system periodically samples stimuli in the environment. Here, we present evidence for rhythmic sampling of internal representations of targets and distractors up to 1 s after visual stimulation offset. Twenty participants performed an anticipatory object-based visual attention task involving a short-lasting (50 ms) target object image. This task was made more challenging by the overlaid presentation of a distractor object image. We conducted a decoding analysis on stimulus-evoked electroencephalography to measure target and distractor information over the stimulus epoch, which extended almost 1,000 ms beyond the visual stimulus offset. We found that the magnitudes of target and distractor information represented in brain activity after the offset of the visual stimuli oscillated in the theta frequency range (4 to 8 Hz). This oscillatory period accords with previous characterizations of rhythmic attentional sampling of continuously visually presented stimuli. Moreover, greater target–distractor theta band phase differences correlated with improved task performance. Our findings show the following: (i) attention separately samples target and distractor representations in sensory memory, (ii) these separately sampled streams of information may mutually inhibit one another, and (iii) target discrimination improves when target and distractor sampling rhythms are desynchronized.

## Introduction

Visual attention is a neurocognitive function that directs the limited processing capacity of the brain’s visual system to information relevant for behavioral goals. While attention has sometimes been viewed as a sustained “spotlight,” or involving a rapid searchlight mechanism (Crick 1984), recent findings suggest that a rhythmic sampling process is involved during sustained attention, often in the theta frequency range of approximately 4 to 8 Hz (Landau and Fries 2012; Fiebelkorn and Kastner 2019; Re et al. 2019). According to this view, attentional selection fluctuates periodically, enhancing the processing of relevant stimuli only during discrete high-excitability phases rather than continuously.

One key piece of evidence for this rhythmic approach is that the detection rate of visual targets can vary periodically with the interval between a cue and a target, in line with cycles of neural excitability (Busch et al. 2009; Fiebelkorn et al. 2013). If the target appears during a low excitability phase, detection performance is worse than when the same target is presented in a high excitatory phase. Such periodicity has been hypothesized to govern not only spatial selection but also feature (Mo et al. 2019; Re et al. 2019) and object-based attention (Fiebelkorn and Kastner 2019), particularly when multiple sources must be sampled in quick succession.

A missing piece in this literature, however, is how internal sampling of sensory input representations operates, especially for target versus distractor competition. It has been shown that attention can be directed toward internal representations of sensory information, such as in sensory memory (Xie et al. 2021) and working memory (van Ede and Nobre 2023), where there is also evidence for rhythmic sampling (Chota et al. 2022). While there is evidence for competition between objects presented together in the visual field (Moldakarimov et al. 2005; Rollenhagen and Olson 2005), it is not known whether representations of targets and distractors in sensory buffers also show rhythmic competitive interactions. Addressing these issues is critical to understanding how the brain manages interference among multiple stimuli in real-world contexts, where relevant and irrelevant objects frequently appear together.

In the present study, we tested the hypothesis that rhythmic sampling can operate over multiple sources of information in parallel—specifically, target detection and distractor suppression—even after the physical stimuli are no longer present in the environment. We performed electroencephalography (EEG) decoding to quantify how target and distractor information persisted after the stimulus offset, and how these representations interacted. Our analysis revealed periodic theta-band (4 to 8 Hz) fluctuations in both target and distractor representations, extending earlier findings on attentional sampling of stimuli when present in the visual field (Fiebelkorn et al. 2013; Thigpen et al. 2019). Importantly, we also observed a correlation between task accuracy and the phase difference of these rhythmic representations across subjects, indicating that when target and distractor were sampled in phase, they appeared to compete and degrade behavioral performance. This finding suggests that attention can rhythmically sample internal representations and that synchronizing these sampling streams results in interference.

Our findings highlight 3 main points: (i) attention separately samples target and distractor representations in visual sensory memory, (ii) these separately sampled streams of information interfere with one another, and (iii) target discrimination improves when target and distractor sampling rhythms are desynchronized or out of phase with each other.

This demonstration of rhythmic sampling between simultaneously presented and overlapping target–distractor objects, even after these stimuli are no longer present in the visual field, advances our understanding of how attentional processes manage competing information sources in everyday vision.

## Materials and methods

In this study, we analyzed data collected as part of work investigating the neural mechanisms of anticipatory object-based visual attention. Detailed methods for the experimental paradigm, EEG preprocessing, and EEG decoding analysis procedure are described in our previous publication (Noah et al. 2023).

### Participants

We recruited 23 healthy volunteers from the University of California, Davis to participate in our object-based attention EEG experiment. All participants had normal or corrected-to-normal vision, were free of neuropsychiatric conditions, gave written informed consent for their participation, and received monetary compensation for their time. This study protocol was approved by the Institutional Review Board of the University of California, Davis (IRB protocol number 219132). We rejected the data of 3 participants due to intractable noise in the EEG data or task noncompliance (eg falling asleep during the experiment, not following task directions), leaving a final EEG data set from 20 participants (9 men and 11 women).

### Task and stimuli

**Figure 1:**
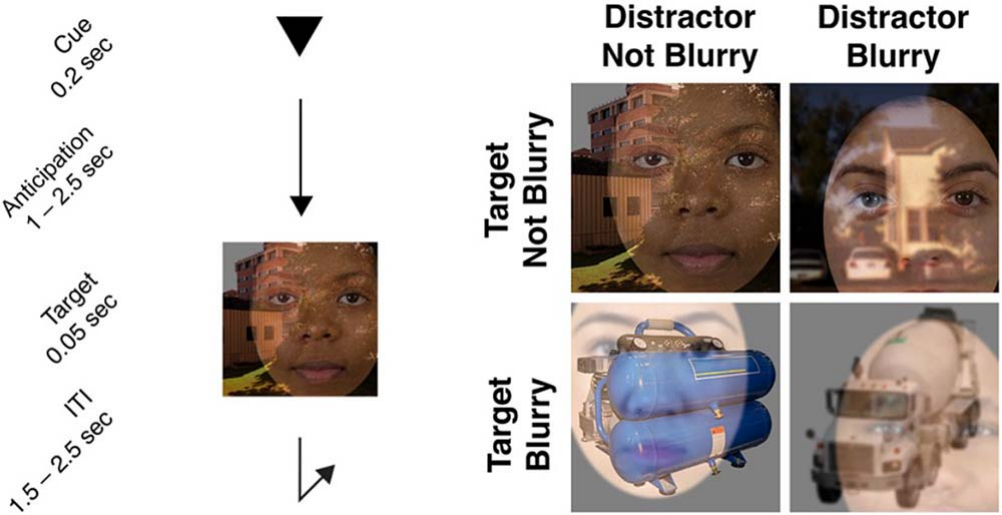
Object-based attention experimental paradigm. Participants were cued to attend to 1 of 3 object categories: faces, scenes, or tools. Stimuli were overlaid target and distractor images. Both the target and distractor could be blurry or not blurry, independently of one another. ITI, inter-trial interval.

Participants performed an anticipatory object-based visual attention task. On each trial, a symbolic shape cue informed participants about the category of an upcoming visual object stimulus (80% validity). The 3 categories of stimuli were faces, scenes, and tools. Stimuli were overlaid images belonging to 2 categories, one of which was the target (cued) category, and the other of which was a distractor. Both the target and distractor images could be either blurry or not blurry, independently of one another. The stimuli were 5° × 5° square images presented on screen for 50 ms (Fig. 1).

The participants were instructed to respond to each stimulus with a button press during the inter-trial interval (ITI) to indicate whether the target object image in the stimulus was blurry or not blurry. Each participant completed 15 blocks of the experiment while EEG was recorded, with each block comprising 42 trials (630 trials total).

On invalid trials, only 1 target image was presented, belonging to an uncued object category, and overlaid with a checkerboard image that could also be either blurry or not blurry. Participants were instructed that for these trials, the perceptual judgment needed to be made and reported on the basis of whatever object image was present in the stimulus, regardless of cued object category.

### E‌EG acquisition and preprocessing

Raw EEG data were acquired with a 64-channel Brain Products actiCAP active electrode system (Brain Products GmbH), amplified and digitized using a Neuroscan SynAmps2 input board and amplifier (Compumedics USA, Inc.), and recorded with Scan 4.5 acquisition software (Compumedics USA, Inc.) at a sampling rate of 1,000 Hz. The electrodes were placed in fitted elastic caps in a montage according to the International 10–10 system (Jurcak et al. 2007).

EEG data were preprocessed with the EEGLAB MATLAB software (Delorme and Makeig 2004). The data were Hamming window sinc finite impulse response filtered (1 to 83 Hz) and down-sampled to 250 Hz. Data were epoched from 500 ms before stimulus onset to 1,000 ms after stimulus onset. Independent component analysis decomposition was used to remove artifacts associated with blinks and eye movements.

### E‌EG decoding analysis

We performed an EEG decoding analysis to quantitatively assess whether target and distractor visual information was systematically associated with changes in phase-locked event-related potential (ERP) voltage topography. Decoding was performed independently at each time point within the stimulus epoch of 500 ms before to 1,000 ms after stimulus onset. We implemented support vector machine classification via the MATLAB *fitcsvm*() function. For every time point, a binary classifier was trained to classify whether single-timepoint ERP data belonged to a trial in which the target stimulus was blurry or not blurry, and a separate binary classifier was trained to classify whether the data belonged to a trial in which the distractor stimulus was blurry or not blurry. For each classification, decoding was considered correct when the classifier correctly determined the target or distractor blurriness from the 2 possible conditions; thus, chance performance was set at 50%.

The decoding procedure resulted in a target decoding accuracy time series and a distractor decoding accuracy time series. Each time series could be interpreted as the amount of information about the target or distractor present in the scalp voltage topography, over time (Bae and Luck 2018; Noah et al. 2020). We only performed the decoding analysis on valid trials because on invalid trials, there were not 2 object images presented.

### Decoding time series spectral analysis

We separated the poststimulus-onset epoch into a 0 to 300 ms period and 300 to 1,000 ms period, based on our previous study suggesting that up to 300 ms poststimulus onset, target and distractor information were matched (Noah et al. 2023). To focus the present analysis on oscillations in and competition between target and distractor representations, we computed the power spectrum for each participant’s target and distractor decoding accuracy time series (a single scalar value per time point reflecting information contained in the multichannel scalp pattern) in the 300 to 1,000 ms range. All spectral analysis was performed using the Python programming language with the SciPy and NumPy software libraries (Harris et al. 2020; Virtanen et al. 2020).

For each participant and condition (target and distractor), the decoding accuracy time series was transformed into the frequency domain using a discrete Fourier transform implemented in NumPy (numpy.fft.fft). Prior to transformation, no additional detrending, tapering, or zero padding was applied; thus, frequency resolution was determined solely by the duration of the analyzed time window and the sampling rate of the decoding time series. Power spectra were computed as the squared magnitude of the complex Fourier coefficients.

Next, we investigated the across-participants correlation between target and distractor theta band phase difference and task performance. For each participant, we calculated the phase at each of the 3 frequencies in our theta band, for both the target and distractor power spectra. Phase information was extracted directly from the complex-valued Fourier coefficients by computing the argument of each coefficient. We computed the phase difference between target and distractor, wrapped to −π and π radians. We converted the phase differences to absolute phase differences because we were specifically interested in the magnitude of the phase difference between targets and distractors, irrespective of whether one time series was ahead of or behind the other. We also calculated the mean phase difference across the 3 frequencies in our theta band. We performed Pearson correlation of these four phase difference values with each individual participant’s behavioral performance, measured by the percentage of trials performed correctly in the behavioral task. For each correlation, we computed a correlation coefficient and a *P*-value for hypothesis testing. We divided our hypothesis test decision criterion by 4 in accordance with the Bonferroni method for multiple comparisons, resulting in a *P*-value threshold of 0.0125 for rejecting the null hypothesis that there was no correlation between target–distractor phase difference and task performance.

Additionally, we compared the theta power in the poststimulus-onset period to the baseline, prestimulus-onset period. For this analysis, theta-band power (4 to 8 Hz) was computed for each participant from the decoding accuracy time series during a prestimulus baseline interval (−500 to 0 ms) and during 2 poststimulus intervals (0 to 1,000 ms and 300 to 1,000 ms). To obtain a single measure of overall decoded-information-related theta activity, theta power was averaged across target and distractor decoding time series within each interval. Baseline and poststimulus theta power were then compared using paired-sample statistical tests, and effect sizes were quantified using Cohen’s dz. In addition, a 2 × 2 repeated-measures analysis of variance (ANOVA) with factors condition (target, distractor) and period (baseline, poststimulus) was conducted to assess whether theta power differed across conditions or time periods.

### Behavioral analysis

To determine whether attention was allocated successfully to target stimuli, behavioral performance was quantified separately for valid and invalid trials and compared. For each participant, mean accuracy and mean reaction time were computed across trials of each validity condition, producing per-participant summary measures. Differences between valid and invalid trials were assessed using paired-sample statistical tests with directional hypotheses, reflecting the a priori expectation that valid trials would be associated with higher accuracy and faster responses than invalid trials. One-tailed paired *t*-tests were conducted, and effect sizes were quantified using Cohen’s dz.

## Results

### Spectral analysis results

**Figure 2:**
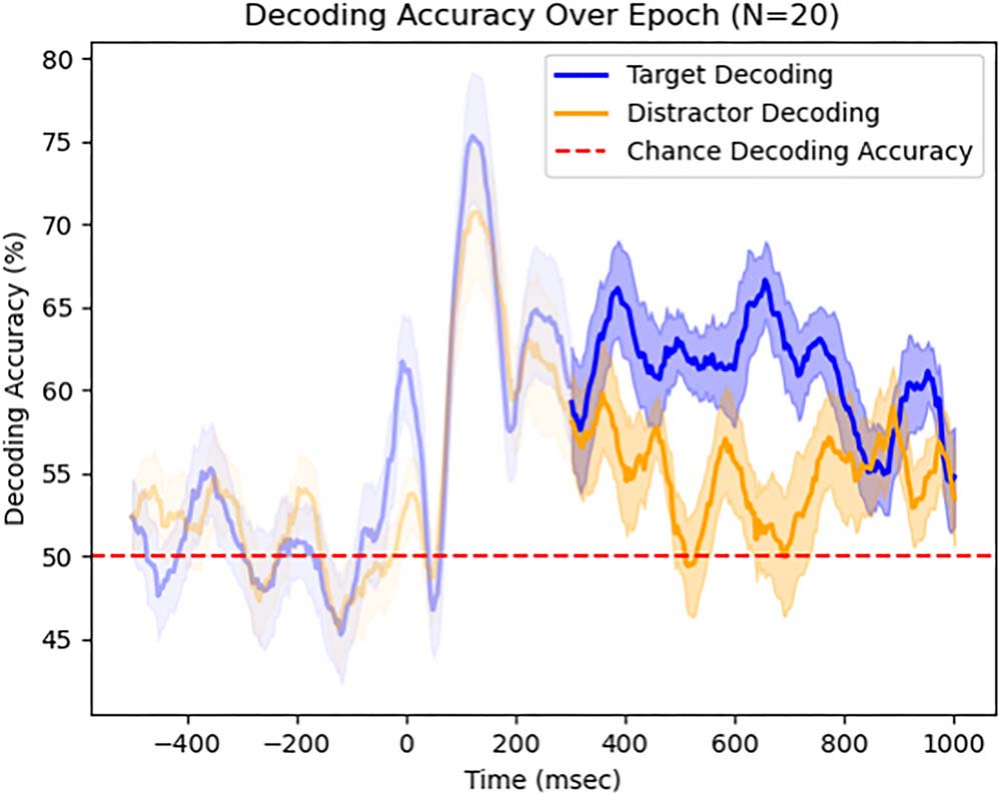
Decoding accuracy time series for targets and distractors. 0 ms marks the time of stimulus onset. The portion of the time series in a more saturated color from 300 to 1,000 ms contains the decoding time series that was passed to subsequent spectral analyses.

**Figure 3:**
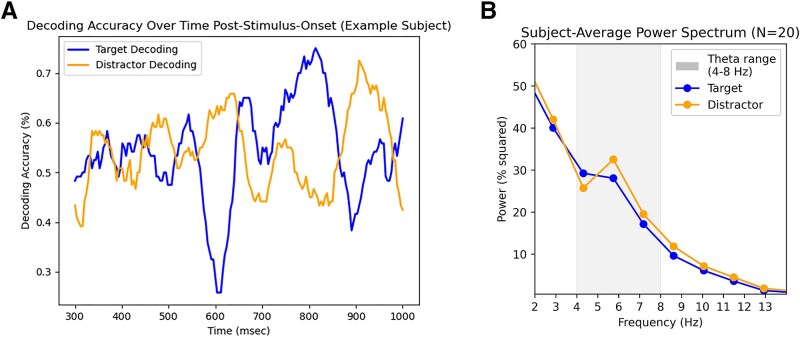
A) Decoding accuracy time series (300 to 1,000 ms) for an individual example participant. B) Average power spectra for target and distractor decoding accuracy time series (300 to 1,000 ms), showing peaks in the theta band frequency range.

**Figure 4:**
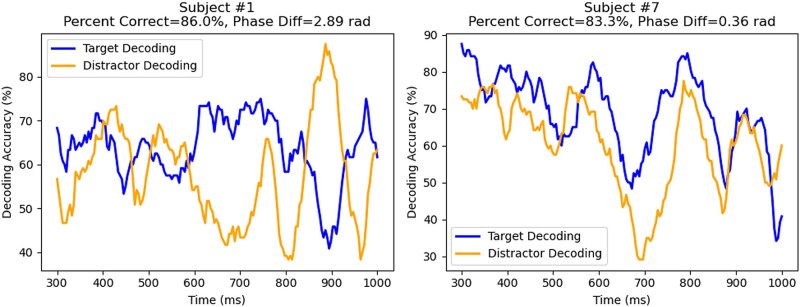
Individual subject target and distractor decoding accuracy time series, showing an example of a subject with a large average phase difference across the 3 theta band frequencies and relatively higher behavioral performance, and a subject with the opposite characteristic.

Target and distractor time series appeared to oscillate in an antiphase relationship (Fig. 2; Fig. 3A; Fig. 4). We performed power spectral analysis on our target and distractor decoding accuracy time series in the 300 to 1,000 ms period to investigate this finding.

Averaging the target and distractor decoding accuracy time series power spectra across participants revealed peaks in the theta band (Fig. 3B). Because of the number of datapoints in our decoding accuracy time series, the theta band in our power spectra comprised 3 frequencies: 4.3, 5.7, and 7.2 Hz. The target time series had greater power than the distractor time series at 4.3 Hz, whereas the distractor time series had greater power at 5.7 and 7.2 Hz.

Additionally, we compared theta power in the poststimulus interval to the prestimulus baseline. Collapsing across target and distractor decoding time series, theta power was significantly elevated in both the 0 to 1,000 ms interval [*t*(19) = 2.50, *P* = 0.022, Cohen’s dz = 0.56] and the 300 to 1,000 ms interval [*t*(19) = 2.22, *P* = 0.039, dz = 0.50] relative to baseline. A complementary 2 × 2 repeated-measures ANOVA with factors condition (target, distractor) and window (baseline, poststimulus) revealed no main effects of condition or window and no interaction for either poststimulus interval (all *P*s > 0.24), indicating that absolute theta power did not differ reliably between target and distractor decoding signals.

### Target–distractor phase difference and behavior correlations

**Figure 5:**
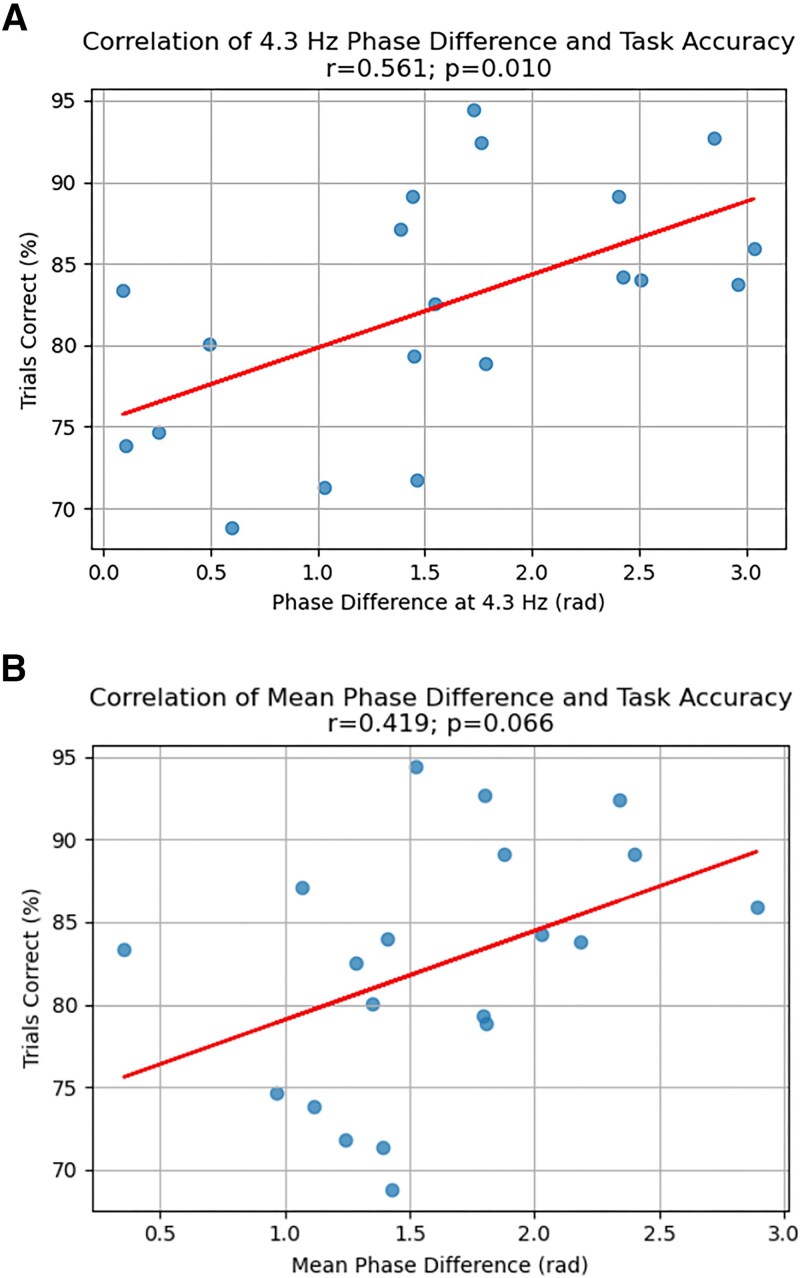
A) Correlation across 20 participants of phase difference at 4.3 Hz between target and distractor decoding accuracy power spectra with percentage of correct trials. In both panels, individual participant data is represented by dots. A regression line fit to the data in each panel is depicted in red. B) Correlation across 20 participants of average phase difference across the 3 frequencies in the theta range of the target and distractor decoding accuracy power spectra with percentage of correct trials.

In our analysis of the association between target–distractor phase difference and task performance, we found positive correlations in all four tests (Fig. 5; Table 1). The correlation of the average theta band phase difference with task performance was positive (*r* = 0.419) but not significant (*P* = 0.066). Of the 3 theta band frequencies in our power spectra, the phase differences at 5.7 and 7.2 Hz were not significantly correlated with task performance. However, the phase difference at 4.3 Hz was significantly correlated with task performance (*P* = 0.010).

**Table 1:** Statistical results for correlation tests. We computed correlation coefficients and *P*-values for correlations between the percentage of task trials correct and the absolute phase difference (0 to π radians), for the 3 individual frequencies constituting the theta band in our power spectra, and the average over the 3 frequencies. Asterisks (^*^) indicate a *P*-value below the threshold of statistical significance after Bonferroni correction for multiple comparisons.

Power spectrum theta band frequency	Correlation coefficient	*P*-value
4.3 Hz	0.561	0.010^*^
5.7 Hz	0.102	0.670
7.2 Hz	0.135	0.572
Average	0.419	0.066

### Behavioral results

**Figure 6:**
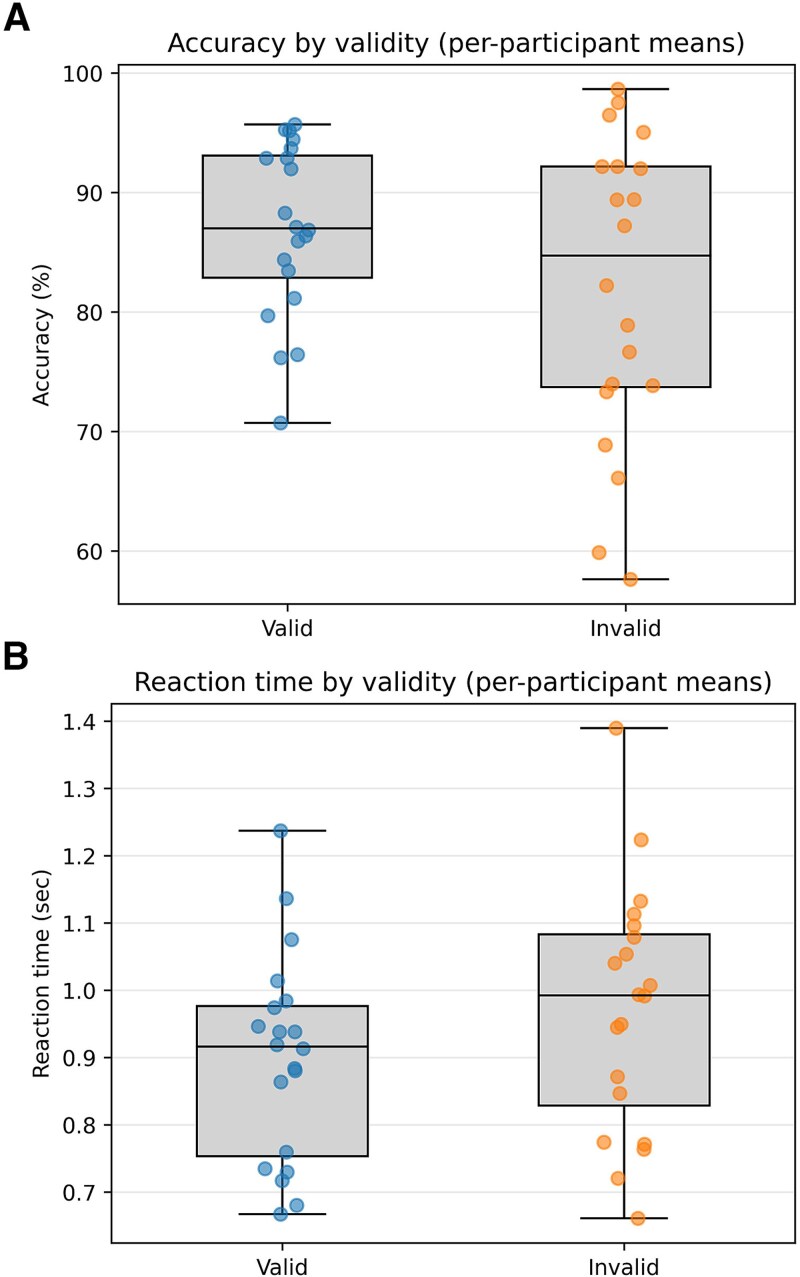
A) Mean accuracy for valid and invalid trials, computed separately for each participant and summarized across the group. B) Mean reaction time for valid and invalid trials, computed separately for each participant. In both panels, box-and-whisker plots summarize the distribution across participants (center line indicates the median; boxes indicate the interquartile range; whiskers extend to 1.5× the interquartile range). Individual participant means are overlaid as points. Valid trials were associated with higher accuracy and faster reaction times than invalid trials, indicating behavioral benefits of cue validity.

Behavioral performance differed reliably between valid and invalid trials (Fig. 6). Accuracy was significantly higher on valid trials than on invalid trials [*t*(19) = 2.36, *P* = 0.015, one-tailed; Cohen’s dz = 0.53], corresponding to an average improvement of approximately 4.9 percentage points. Reaction times were also significantly faster on valid trials [t(19) = −4.96, *P* < 0.001, one-tailed; Cohen’s dz = −1.11]. These results suggest that cue validity improved behavioral performance across participants.

## Discussion

Attention has been characterized as a rhythmic sampling process (Landau and Fries 2012; Fiebelkorn and Kastner 2019; Re et al. 2019). In this view, rhythmic sampling is part of the mechanism by which attention divides the brain’s limited processing capacity among multiple task-relevant information sources. At the level of neural implementation, the excitability of neural populations sensitive to disparate visual information sources may fluctuate periodically, and if a task-relevant stimulus appears during a peak in this periodic function, its representation is more likely to influence higher-order cognitive operations (Fiebelkorn and Kastner 2019). Phase-dependent stimulus processing has been inferred from behavioral measurements of task performance, including target detection percentage and reaction time to target. Targets that occur during presumed high-excitability phases of the attentional rhythm elicit faster and more accurate responses (Fiebelkorn et al. 2013).

Moreover, the frequency of this sampling function appears to depend on the number of items being sampled, in line with models of visual processing in which separate items are processed serially rather than in parallel (Thigpen et al. 2019). In 1 experiment, the detectability of a target that could only appear at 1 location varied with a period of around 8 Hz, but when a target could appear at 1 of 2 locations, its detectability varied with a period of around 4 Hz (Fiebelkorn et al. 2013). This proportional relationship between the number of to-be-attended locations and the period of the detectability function has been interpreted as evidence for a single attentional sampling process with capacity limited by the need to sample all to-be-attended locations serially.

A hypothetical single, serial attentional sampling process is difficult to generalize to naturalistic behavior. Natural environments are replete with competing and distracting stimuli, and in many situations, task-relevant objects can appear at any location in the visual field. For example, successfully driving down a busy city street at high speed requires vigilance for the sudden appearance of pedestrians, bicyclists, cars, animals, and obstacles that can appear unexpectedly in many locations, under conditions of ubiquitous salient distractors. While single-mechanism serial sampling has strong support in constrained laboratory tasks, the complexity of natural settings—where relevant events may appear anywhere and multiple distractors compete simultaneously—demands a more flexible, parallel, or interleaved sampling architecture.

However, despite the relevance of target–distractor interactions to the question of attentional sampling in natural settings, only 1 study has examined the rhythmic sampling dynamics of simultaneously occurring targets and distractors (Xiong et al. 2024). Our study adds to this body of work and offers an alternative theoretical account of rhythmic attentional behavior.

A possible alternative to the single, serial attentional sampling hypothesis is that rhythmicity is an intrinsic characteristic of neural representations of sensory information. According to this view, neural representations of disparate sensory stimuli compete for access to higher-order cognitive operations, and periodic fluctuations in the strength of the stimulus representations are part of the mechanism by which limited processing resources are divided among multiple possibly task-relevant information sources. This hypothesis implies that when 2 separate representations are fluctuating in phase, they would interfere with each other and hinder higher-order cognitive operations that make use of these representations.

We found that target and distractor decoding accuracy time series fluctuate periodically with a peak in the theta range of 4 to 8 Hz. Furthermore, we found a statistically significant across-participants correlation between the target–distractor decoding time series phase difference at 4.3 Hz and behavioral accuracy on a target discrimination task. Our theoretical interpretations of our findings are (i) attention separately samples target and distractor representations during the cognitive processes that occur between stimulus presentation and behavioral response, (ii) these separately sampled streams of information interfere with one another, and (iii) target discrimination improves when target and distractor sampling rhythms are desynchronized. These interpretations may motivate future research, such as to investigate causal mechanisms of the mutual inhibition of target and distractor representations.

Previous work has provided evidence for sampling of internal representations of sensory information. For example, internal working memory representations of sensory information are subject to a sampling-like process during decision-making (van Ede and Nobre 2024). Attention can operate over internal representations of sensory information, including in sensory memory (Xie et al. 2021) and working memory (van Ede and Nobre 2023). There is also behavioral evidence for rhythmic sampling in working memory (Chota et al. 2022). However, our study is the first to demonstrate rhythmic attentional sampling of internal stimulus representations in neural activity. The visual stimuli in our experimental paradigm were presented for 50 ms, and any target or distractor stimulus information that could be decoded from EEG after this interval therefore belonged to an internal representation of the visual stimulus. We showed that periodically fluctuating stimulus representations were present in cortical activity for up to 1 s after stimulus onset, during the time when participants were preparing their behavioral responses.

Previous studies of rhythmic attentional sampling have shown a periodic relationship between behavioral measures of target detection and cue-to-target stimulus onset asynchrony. Our observation of periodic fluctuations in stimulus decoding accuracy suggests that the attentional elaboration of internal information is subject to the same rhythmic attentional processes. Moreover, this finding suggests that rhythmic fluctuations in representation strength are a characteristic of neural computation that is more fundamental than sensory sampling.

We computed the correlation between the phase difference of target and distractor decoding accuracy time series and target discrimination task performance across subjects. This correlation was statistically significant for 4.3 Hz fluctuations in target and distractor representation strength. We interpret this correlation to mean that target and distractor representations fluctuate separately, and optimal task performance occurs when the periodic representations are interleaved out of phase.

In our decoding accuracy time series for targets and distractors, we observed periodic fluctuations 300 to 1,000 ms poststimulus onset that appear to be largely antiphase (Fig. 2). In previous work, we showed that there is a statistically significant anticorrelation between target and distractor decoding accuracy timepoints in this time period (Noah et al. 2023). In the present article, we report a correlation between target–distractor theta band phase difference and target detection task performance. We hypothesize that this antiphase relationship between target and distractor representations results from top-down attentional mechanisms that strive to interleave competing stimulus representations according to task demands. This kind of active phase adjustment mechanism presents an alternative explanation to a serial sampling mechanism that has been theorized as an explanation for antiphase relationships between effective target processing at multiple locations. Attentional interleaving that varies in efficacy across trials or across individuals would explain why a distribution of phase differences at the proposed sampling frequency was observed across participants in our study. Future studies can be designed to clarify this hypothetical attentional interleaving mechanism.
